# It’s All about Those Bases: The Need for Incorporating Parasite Genetic Heterogeneity into the Development of Schistosome Vaccines

**DOI:** 10.1371/journal.pntd.0003805

**Published:** 2015-06-18

**Authors:** Alyssa M. Gleichsner, Elizabeth A. Thiele, Dennis J. Minchella

**Affiliations:** 1 Department of Biological Sciences, Purdue University, West Lafayette, Indiana, United States of America; 2 Department of Biology, Vassar College, Poughkeepsie, New York, United States of America; McGill University, CANADA

Parasite genetic variation has been used to delineate and enumerate species, describe host–parasite associations, and observe the distribution of lineages among hosts and throughout the environment. Mechanisms or pathways inferred by these studies have often been carried forward in a predictive manner to inform assumptions about the organism’s biology, its life history, and host–parasite interactions. For medically relevant parasites, these observations have undoubtedly proven useful for a fuller epidemiological understanding of parasite transmission pathways and patterns. Here, we argue that functional parasite genetic variation in natural populations of schistosomes is poorly understood and that a better understanding of this variation is crucial to the development of effective vaccines.

Parasites in the genus *Schistosoma* are responsible for causing the disease schistosomiasis, a neglected tropical disease that affects over 200 million people in 74 countries [1]. Despite considerable effort and exploration, an effective human vaccine for this disease remains elusive [1]. An increasing array of studies assessing genetic variation within the medically relevant species of *Schistosoma* has indicated that old paradigms of focal transmission patterns and subsequently restricted parasite gene flow do not always hold up against genetic evidence (e.g., [2,3]). These conclusions are generated by the often surprising amount of genetic variation observed within natural schistosome populations, where even within the limited radius of a single village, high neutral genetic variation has been maintained [2]. How universal this is, how it varies across landscapes—both biological and cultural—and what it implies for the host–parasite coevolutionary trajectory has yet to be fully explored. However, it is clear that a remarkable amount of genetic variation has been recorded within natural populations of schistosome species. These observations have been largely generated with presumed neutral, noncoding loci like microsatellites [2,4] and random amplified polymorphic DNAs (RAPDs) or with mitochondrial haplotypes [3]—none of which are expressly indicative of variation in functional regions of the genome. Yet, even a minimal connection between neutral genetic variation and the functional end of the “-ome” should perhaps be concerning to scientists focused on developing an effective schistosome vaccine with long-lasting potential.

Unfortunately, to date, much of schistosome genomics and molecular biology, as well as the vast majority of studies assessing vaccine efficacy, have used laboratory strains of parasites both to develop antigenic proteins and as the experimental subject. A review of 120 schistosome vaccine studies published in the past 7 years (using the search terms: schistosom*, vaccine*, 2007–2015) and queries of GenBank sequence repositories for key terms typically seen in the vaccine literature (e.g., tegument, tetraspanin, glycan, etc.) found that laboratory strains were dramatically over-represented relative to field strains for all *Schistosoma mansoni* studies and in submitted sequence data (Table 1). Within *S*. *japonicum*, the Chinese mainland strain is used disproportionally to other regional isolates. These parasite lines, in particular those of *S*. *mansoni*, have been maintained in limited population sizes for up to 100 or more generations [5]. Comparison to field parasite populations [6] and longitudinal evaluations of neutral genetic variation [7] indicate that the effects of a laboratory bottleneck can be significant and rapid. In short, much of our-omics and empirical investigations of biomolecular host–parasite interaction are based on what could effectively be a genetically limited caricature of the parasite we are trying to prevent.

**Table 1:** Percentage of published schistosome vaccination studies and GenBank genomic and expressed sequence tag (EST) entries from 2007–present that utilized laboratory or field isolates.

*Schistosoma* spp.		Published Experiments (*n* = 121)		GenBank Genomic and EST Entries (*n* = 1,409)
		Parasite	Host	Parasite
***S*. *mansoni***	Lab strain	80%	93%	79%
	Field	8%	7%	2%
	Not specified	12%	0%	19%
***S*. *japonicum***	Chinese mainland	74%	87%	84%
	Other	12%	10%	6%
	Not specified	14%	3%	10%

Results indicate that the majority of experiments use laboratory-maintained parasites and hosts.

Granted, we expect high levels of polymorphism and rapid change in neutral alleles like microsatellites. Yet one could also argue, and several have demonstrated, that schistosomes have surprising potential for generating variation in functional regions as well. These variations include single-nucleotide polymorphisms in coding regions that can produce significant conformational changes in proteins [8], impressive capacity for variation in proteins at the host–parasite interface due to post-translational modification and alternative splicing [9], and an increasingly appreciated role of transposable elements in generating and maintaining variation throughout the genome [10]. We do not suggest that the entire assemblage of functional regions in the schistosome genome is likely to be hypervariable or easily mutable. However, the observations referenced here are noteworthy in that they have documented variation in coding regions that are most often targeted for vaccine research. These are often tegumental moieties (e.g., [11–13]) but in general represent anything at the host–parasite interface that is likely to elicit an immune response for which the human host can be primed via inoculation. As is also pointed out by Philippsen et al. [10], these are the exact entities that one would expect to be under intense evolutionary pressure to maintain diversifying potential in a blood-dwelling parasite that seeks to evade immune detection by the highly variable vertebrate immune system. Thus, it is perhaps not surprising that the few studies that have looked at these areas have found high variation. Different types of vaccine targets, such as internal proteins, may not be under such strong diversifying selective pressure, but, again, this is a question that remains largely unaddressed. In short, our general lack of understanding about the full extent and distribution of variation throughout the genome, and in the majority of vaccine target regions, is a problem that schistosome researchers have not yet addressed. Other high-profile diseases have faced difficulties concerning vaccine development and high pathogen variability (e.g., malaria, HIV, tuberculosis, and hepatitis C), suggesting that researchers working towards the production of schistosome vaccines should be concerned not only with extant genetic variability but with the parasite’s ability to generate variation.

The deficiency of studies examining genetic diversity in recent field isolates at non-neutral sites (e.g., those encoding tegument proteins) presents a gap in our knowledge about schistosome populations, one that could directly impact the success of human vaccines implemented into the field. This viewpoint should in no way be considered a rebuke to the schistosome community, but rather a call to acknowledge and seek to better understand the breadth of the parasitic arsenal in this particular arms race. We specifically acknowledge the logistical difficulty associated with acquiring parasites from the field and the daunting financial requirements for the-omics work that has been done to date and has yet to be done. Moreover, apart from logistics and finances, development of appropriately robust references for each species required access to a single well-characterized lineage with ample biological resources. But now, with reference genomes [14–17], transcriptomes [15,18–20], exomes [21], phylogenomes [22], and proteomes [19,20,23] in hand, there exist a variety of resources that increase the ability of researchers to examine this variation. While not reflective of field variation, these resources vastly increase the ease of-omics work by providing a means for the creation of genetic tools [4] that can then be used in the field and a template that researchers can use to map their data. Aside from field collection, existing museum collections can be put to use—for example, the cercariae bank SCAN (Schistosomiasis Collection at the Natural History Museum–London) [24], from which researchers can obtain cercariae samples from several geographic locations to investigate genetic variability. These resources will allow researchers to check levels of variation within target genes of interest (which may be the most cost-effective and efficient approach) and begin to understand adaptive variation on a genome-wide and global scale.

Likewise, the continued advances in next-generation sequencing and analysis have exponentially increased our ability to generate genome-wide data with high and affordable throughput. Though they might not move at the pace of biotechnology, the bioinformatics and statistical analytics to accompany the data generation are also making giant strides. Methods such as whole-genome sequencing of pooled DNA samples (Pool-Seq), already used on laboratory populations of *S*. *mansoni* to evaluate variation across the genome [5], could be used to interrogate larger swaths of field-collected specimens across the whole genome without the expense of sequencing multiple specimens individually. Together with an increasingly annotated and functionally described genome, these data could go a long way to both providing an increased understanding of the schistosome’s biology and ensuring a rationally devised and informed suite of human vaccine targets that incorporates, a priori, the parasite’s potential to adapt.

Schistosomes present a clear burden of disease on much of the globe’s population. We know from current data that these parasites can harbor significant amounts of genetic variation, but we are only just beginning to understand how that variation could alter approaches to vaccine development. Gobert et al. [25] recently called for an increased effort to place schistosome genomic elements into a more biologically functional perspective. We build upon this call and encourage the schistosome community to incorporate studies that will provide perspective on the variable nature of the schistosome’s functional biology. As we explore the target(s) that will most effectively elicit a protective immune response, we cannot overlook the role that natural adaptive variation of the parasite could play in thwarting widespread vaccine efficacy.
